# Complete Chloroplast Genome Sequence of *Fagus longipetiolata* Seemen (Fagaceae): Genome Structure, Adaptive Evolution, and Phylogenetic Relationships

**DOI:** 10.3390/life12010092

**Published:** 2022-01-09

**Authors:** Daqu Liang, Haoyun Wang, Jun Zhang, Yuanxiang Zhao, Feng Wu

**Affiliations:** Institute for Forest Resources & Environment of Guizhou, Key Laboratory of Forest Cultivation in Plateau Mountain of Guizhou Province, College of Forestry, Guizhou University, Guiyang 550025, China; liangdaqu66@163.com (D.L.); GZhaoyunwang@126.com (H.W.); zhangjun20210802@163.com (J.Z.); yxzhao0602@163.com (Y.Z.)

**Keywords:** *F. longipetiolata*, chloroplast genome, comparative analysis, purifying selection, phylogenetic analysis

## Abstract

*Fagus longipetiolata* Seemen is a deciduous tree of the *Fagus* genus in Fagaceae, which is endemic to China. In this study, we successfully sequenced the cp genome of *F. longipetiolata*, compared the cp genomes of the *Fagus* genus, and reconstructed the phylogeny of Fagaceae. The results showed that the cp genome of *F. longipetiolata* was 158,350 bp, including a pair of inverted repeat (IRA and IRB) regions with a length of 25,894 bp each, a large single-copy (LSC) region of 87,671 bp, and a small single-copy (SSC) region of 18,891 bp. The genome encoded 131 unique genes, including 81 protein-coding genes, 37 transfer RNA genes (tRNAs), 8 ribosomal RNA genes (rRNAs), and 5 pseudogenes. In addition, 33 codons and 258 simple sequence repeats (SSRs) were identified. The cp genomes of *Fagus* were relatively conserved, especially the IR regions, which showed the best conservation, and no inversions or rearrangements were found. The five regions with the largest variations were the *rps12*, *rpl32*, *ccsA*, *trnW-CCA,* and *rps3* genes, which spread over in LSC and SSC. The comparison of gene selection pressure indicated that purifying selection was the main selective pattern maintaining important biological functions in *Fagus* cp genomes. However, the *ndhD*, *rpoA*, and *ndhF* genes of *F. longipetiolata* were affected by positive selection. Phylogenetic analysis revealed that *F. longipetiolata* and *F. engleriana* formed a close relationship, which partially overlapped in their distribution in China. Our analysis of the cp genome of *F. longipetiolata* would provide important genetic information for further research into the classification, phylogeny and evolution of *Fagus*.

## 1. Introduction

The *Fagus* genus is mainly composed of deciduous trees and is a genus of the Fagaceae family [1]. *Fagus* species have important economic value and are important components of temperate and subtropical deciduous broad-leaved forests in the Northern Hemisphere; there are approximately 10–13 species of the genus worldwide, including five to seven species found in China [2,3]. Because *Fagus* tree species are widely distributed around Eurasia, the study of the evolutionary history of the genus is conducive to revealing the impact of global climate change on vegetation, as well as the geological and biological connections of this group [4]. Most of the previous studies on the classification of *Fagus* were based on external morphological characteristics, such as the total stem length, involucre bract type and shape, and leaf textural and structural characteristics [5,6]. However, *Fagus* is a relatively specialized group with a rich diversity and complex genetic relationships [7]. The external morphological characteristics of the members of the genus *Fagus* often vary greatly under the influence of environmental factors, and some characteristics are crossed, leading to confusion in their classification [5]. There is still a debate about the number of species in the genus.

In recent years, genome sequencing has been widely used to analyze the genetic variability and evolution of species [8]. Chloroplasts (cp), mitochondria, and nuclei contain independent genomes, which can provide important genetic information for phylogenetic analysis [9]. Chloroplast cells are unique plant cells and the main site of photosynthesis; they have a complete cp genome independent of the nuclear genome [10]. The cp genome is maternally dependent and presents the advantages of a shorter length, easier extraction and purification, a highly conserved sequence and a large number of simple sequence repeat (SSR) loci [11]. When compared with the mitochondrial genome, variations in cp genome size in different plants are relatively low (the mitochondrial genome size of most angiosperms is 300–600 kb, and the cp genome size is 115–165 kb) [12]. Therefore, the cp genome has more important reference value for revealing the origin and evolution of species and determining the genetic relationships between different species. Thus far, the cp genomes of *Fagus engleriana*, *Fagus sylvatica*, *Fagus crenata,* and *Fagus japonica* var. *multinervis* have been published [13,14,15,16]. However, no complete genome sequence of *Fagus longipetiolata*, the *Fagus* species with the most widespread distribution in China [17], has been reported.

In this study, we reported the whole chloroplast genome of *F. longipetiolata* and compared it with the published chloroplast genome of *Fagus*. Moreover, its annotations, codon bias, scattered repeat sequences, and SSRs were analyzed. Our data will be a valuable genetic resource for the study of *Fagus* species.

## 2. Materials and Methods

### 2.1. Sampling, DNA Extraction and Genome Sequencing

Leaves were taken from the *F. longipetiolata* seedlings cultivated at Guizhou University, Guizhou Province, China (26°4.504′ N, 106°6.568′ E), and lodged a voucher specimen (accession number FL-GZU-001) in the Institute for Forest Resources & Environment of Guizhou at Guizhou.

A Plant Genomic DNA Kit (TIANGEN, Beijing, China) was used to extract total genomic DNA from 100 mg of the leaves. The purified DNA was then fragmented by mechanical disruption (sonication). Then, the paired-end (PE) library was constructed using VAHTS Multiplex Oligos set 4 for Illumina (Vazyme, Nanjing, China) and VAHTS Universal DNA Library Prep Kit for Illumina V3 (Vazyme, Nanjing, China), according to the manufacturer’s protocols. Finally, the qualified libraries were sequenced on the Illumina platform, according to the paired-end PE150 sequencing strategy. Approximately 6 Gb of raw data were sequenced. All of the above works were conducted by Genepioneer Biotechnologies Co. Ltd. (Nanjing, China).

### 2.2. Initial Assembly and Annotation of the cp Genome

The cp genome of *F. longipetiolata* was assembled using SPAdes software (v3.10.1) [18] with k-mers of 55, 87, and 121, and the assembly was independent of the reference genome. After assembly, quality control was carried out according to the sequence of *F. japonica* (accession no. MT762295) [19]. We used two methods to annotate the cp genome to improve the accuracy of annotation. First, Prodigal software (v2.6.3) was used to annotate the cp coding sequences (CDSs), Hmmer software (v3.1b2) was used to predict ribosomal RNAs (rRNAs), and Aragorn software (v1.2.38) was used to predict transfer RNAs (tRNAs). Second, the gene sequences were extracted according to the sequences of related species already published in NCBI, and BLAST v2.6 was used to compare the assembled sequence to obtain the second annotation result. Then, the results of the two annotations for different genes were checked manually, erroneous and redundant annotations were removed, and multiexon boundaries were determined to obtain the final annotation. We then mapped the entire genome using OGDRAW software [20].

### 2.3. Codon Usage and Repeat Sequence Analysis

According to the CDSs of 81 protein-coding genes, unique CDSs (one copy selected for CDSs with multiple copies) were screened by using Perl scripts, and the RSCU of each codon was estimated with CodonW 1.4.2 software [21].

The forward repeat sequences, reverse repeat sequences, complementary repeat sequences, and palindromic repeat sequences were analyzed by online Vmatch v2.3.0 software and Perl scripts, with a minimal repeat size of 30 bp and a Hamming distance of 3. MISA v1.0 software was used to search for SSR markers in the cp genome, setting the minimum number of mononucleotide repeats to 8, the minimum number of dinucleotide repeats to 5, and the minimum number of trinucleotide, tetranucleotide, pentanucleotide, and hexanucleotide SSR repeats to 3 [22].

### 2.4. Genome Comparison

Four reported cp genomes of *Fagus* and one exogenous species were downloaded from the NCBI database (*F. sylvatica* (MK598696), *F. engleriana* (KX852398), *F. crenata* (MH171101), *F. japonica* var. *multinervis* (MN894556), and *Arabidopsis thaliana* (AP000423)). The cp genome structures of six plants were analyzed with CGView software [23]. The homology and collinearity of the cp sequences were analyzed with Mauve software [24]. MAFFT software (--auto mode) [25] was used for a global comparison of homologous gene sequences of different plants. DNAsp 5.0 [26] was used to calculate the Pi value of each gene. The boundary information of inverted repeat (IR), small single-copy (SSC), and large single-copy (LSC) regions was visualized by using the SVG module in Perl.

### 2.5. Adaptive Evolution and Phylogenetic Analyses

Based on the five cp genomes of *Fagus* used in this study, the Ka/Ks values of each functional protein-coding gene were calculated by KaKs_Calculator v2.0 software with the default settings [27].

We constructed a phylogenetic tree with the newly sequenced *F. longipetiolata* cp genome and 19 cp genomes (from 1 family, 5 tribes, and 1 outgroup (*Populus trichocarpa*)) downloaded from NCBI. MAFFT (v7.427, --auto mode) was used for multiple sequence alignment [25]. Then, the aligned data were analyzed by RAxML v8.2.10 with the maximum likelihood (MJ) method to construct a phylogenetic tree (1000 bootstraps) [28,29].

## 3. Results

### 3.1. Features of the F. longipetiolata Chloroplast Genome

**Figure 1:**
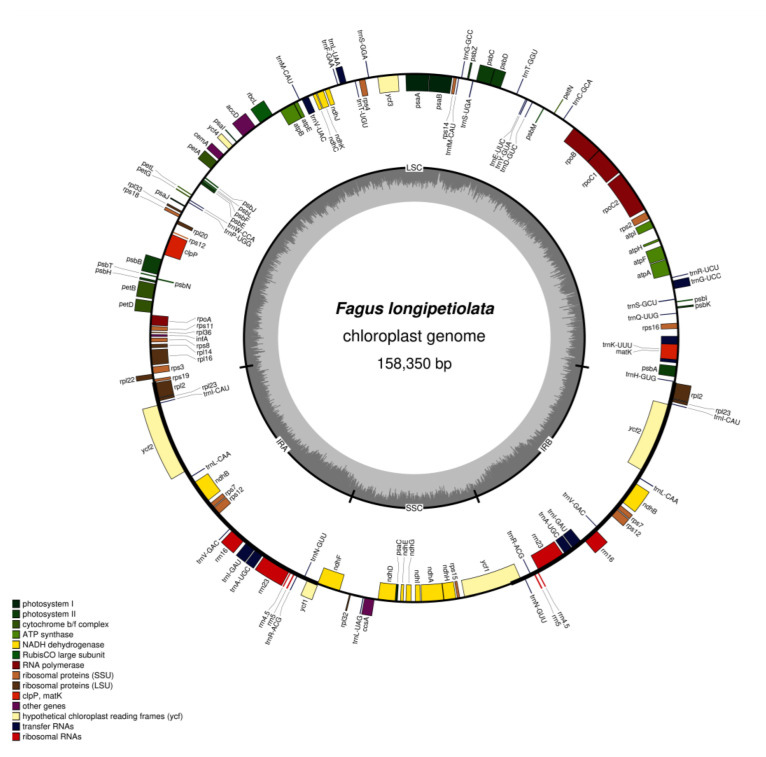
Gene map of the *F. longipetiolata* cp genome. The forward coding genes are located on the outside of the circle, and the reverse coding genes are located on the inside of the circle. Genes with different functions are colour-coded. The deeper grey in the inner circle represents the GC content, while the lighter grey represents the AT content.

A total of 20,928,581 paired-end reads were obtained from the Illumina NovaSeq platform, and the Q20 and Q30 values were 97.53 and 92.98%, respectively. The complete cp genome sequence of *F. longipetiolata* was assembled de novo and uploaded to the NCBI database (GenBank accession number MZ562567). The cp genome of *F. longipetiolata* was a 158,350 bp long circular genome, including a pair of reverse repeats, IRA and IRB (25,894 bp), a small single copy region (SSC, 18,891 bp), and a large single copy region (LSC, 87,671 bp) (Table 1 and Figure 1). The GC content of the IR region sequence was the highest (42.70%), while the GC content of the SSC region was the lowest (31.19%). The average GC content of the whole genome was 37.09%. There were 131 predicted functional genes in the *F. longipetiolata* cp genome, including 81 protein-coding genes, 37 tRNA genes, 8 rRNA genes, and 5 pseudogenes.

Subsequently, we annotated the assembled genes, and all the genes were annotated with gene functions (Table 2). These genes belonged to four types: photosynthesis-related; self-replication-related; genes of unknown function; and maturase (*matK*), protease (*clpP*), and other genes. A total of 18 of the annotated genes were double-copy genes, including three protein-coding genes, seven tRNAs, and eight rRNAs. Fifteen genes (*ndhB*, *petB*, *petD*, *ndhA*, *atpF*, *trnK-UUU*, *trnL-UAA*, *trnA-UGC*, *rpl16*, *rpl2*, *rps12*, *rpoC1*, *trnG-UCC*, *trnI-GAU*, and *trnV-UAC*) had one intron each, and two genes (*ycf3, clpP*) comprised two introns each (Table 2 and Table S1). The longest intron (2524 bp) was located in the *turnK-UUU* gene, which completely encompassed the *matK* gene, and the smallest intron (535 bp) was found in the *trnL-UAA* gene.

### 3.2. Codon Usage Bias

**Figure 2:**
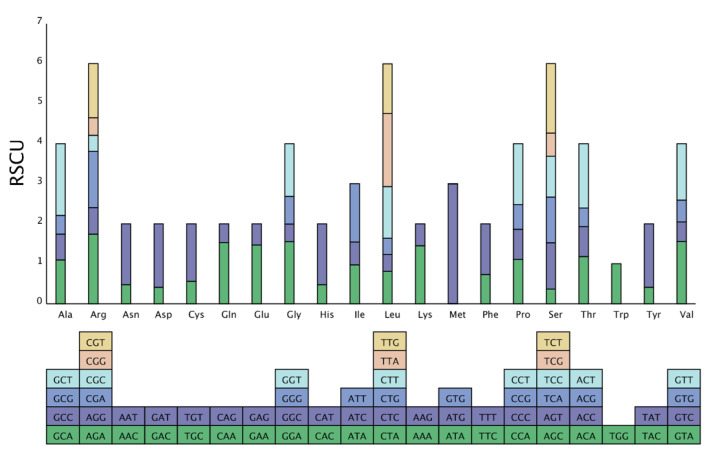
Counting of relative synonymous codon usage (RSCU) of amino acids in the chloroplast genome of *F. longipetiolata*. The colors of the histogram correspond to the colors of the codons.

There are great differences in codon usage among different species and organisms because each amino acid corresponds to at least one codon and, at most, six codons [30]. This inequality of synonymous codon usage is referred to as codon preference (RSCU) [31,32]. Natural selection, species mutation, and genetic drift are considered to be the reasons for this preference [33,34,35]. We screened the unique CDSs and calculated the codon preference (Figure 2 and Table S2). The results showed that the CDS of the *F. longipetiolata* cp genome encoded a total of 24,169 amino acids (including stop codons). Leucine was the most abundant, with 2553 codons (10.56%) detected, followed by isoleucine (2108 codons, 8.72%), and serine (1863 codons, 7.71%), while the rarest amino acid was cysteine (276 codons, 1.14%). Among the codons, 33 (60.94%) preferred codons (RSCU > 1). Twenty-nine preferred codons ended in A/U, but the most preferred codon was AUG, encoding methionine (Met), with an RSCU value of 2.9892.

### 3.3. Detection of Chloroplast Repeat Sequences and SSRs

**Figure 3:**
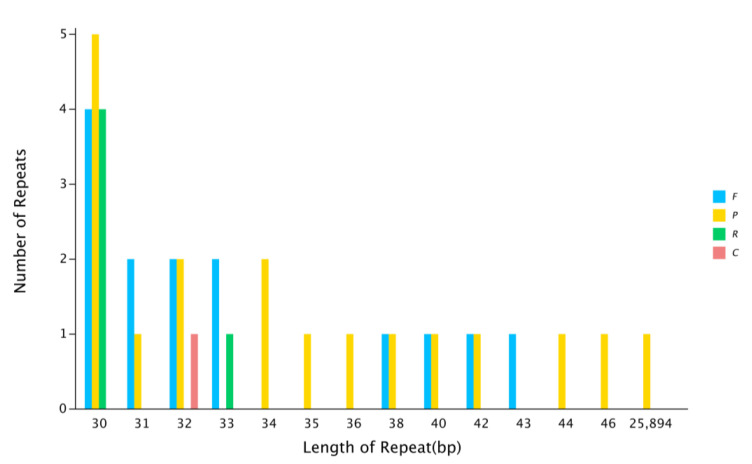
Repeated sequences of the *F. longipetiolata* cp genome. The abscissa is the length of the repeat sequence, and the ordinate is the number of repeat sequences. F stands for forward repetition, P for palindromic repetition, R for reverse repetition, and C for complementary repetition.

In the *F. longipetiolata* cp genome, we discovered 37 repeat sequences. Palindromic repeats were the most common type (17 repeats), accounting for 45.95% of all the repeats, followed by forward (14 repeats, approximately 37.84%), reverse (five repeats, approximately 13.51%), and complementary (one repeat, approximately 2.70%) repeats (Figure 3 and Table S3). A total of 30 repeats were between 30–38 bp in length, and the other repeats were within 40–46 bp. The LSC region had the greatest number of repetitions (26 repeats), followed by the IRs (16 repeats), and the SSC region (seven repeats). In addition, most of the repeats were located in genes (24, 64.87%), and a minority were found in intergenic spacer regions (21, 56.76%).

**Figure 4:**
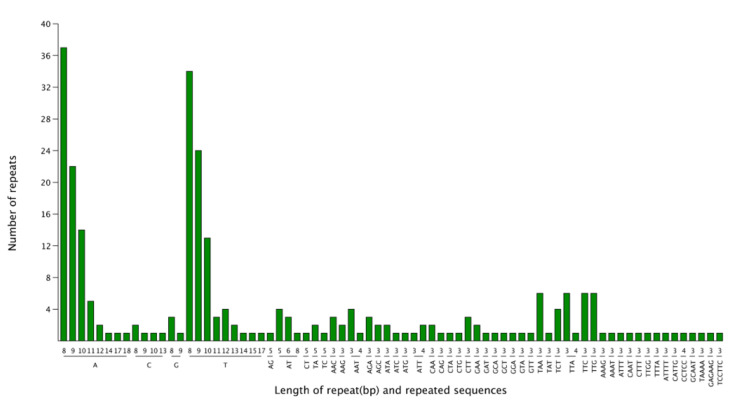
Counting of the types of SSRs. The abscissa represents SSR repetition units, and the ordinate represents the number of SSRs of each type.

We detected 258 SSR loci in the *F. longipetiolata* cp genome. The SSRs were largely distributed in the LSC region (176, 68.22%), followed by the IR regions (46 SSRs), and the SSC region (36 SSRs) (Figure 4 and Table S4). Additionally, 159 SSRs were located in intergenic spaces, and 99 SSRs were located in genes such as *matK*, *atpF*, *trnG-UCC*, *trnK-UUU*, *atpI*, *rpoB*, *rpoC2*, *rpoC1*, *psbC*, *rps14*, *psaB*, *psaA*, *ycf3*, *trnL-UAA,* and *ndhK*. The SSRs consisted of 12 complex nucleotide repeats, 156 mononucleotides, 12 dinucleotides, 66 trinucleotides, 5 tetranucleotides, 5 pentanucleotides, and 2 hexanucleotides, overall. Polyadenine (poly A) and polythymine (poly T) repetitions made up the majority of mononucleotide SSRs (95.51%), whereas C and G mononucleotides were uncommon (4.49%). The larger proportion of A or T bases in certain cpSSRs corresponded to the total A/T content (62.91%) of the *F. longipetiolata* cp genome.

### 3.4. Comparison of Complete Chloroplast Genomes

**Figure 5:**
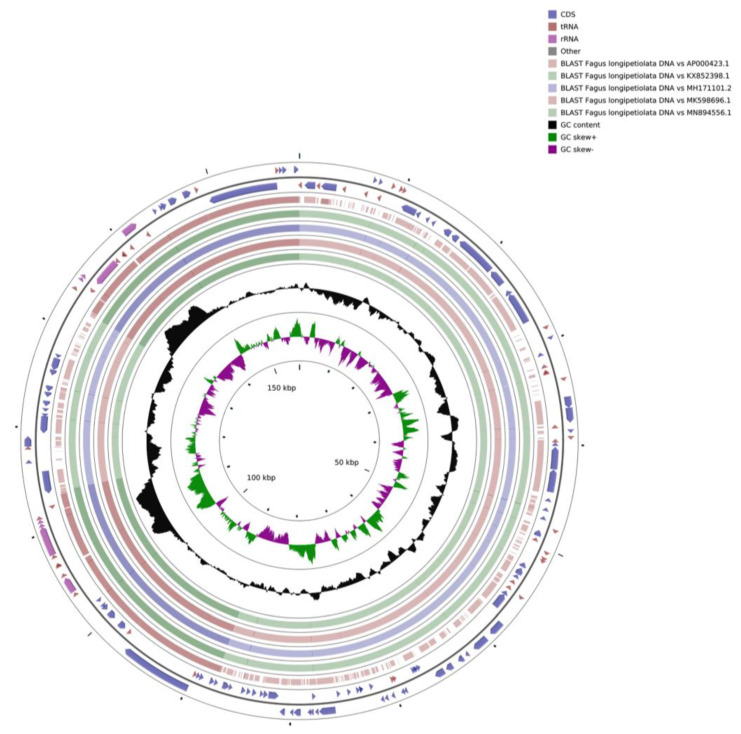
Comparative analysis of cp genome structure between *Fagus* and *A. thaliana*. The outer two circles describe the gene length and direction of the genome. The five circles inside represent the similarity results of genome alignment between *F. longipetiolata* and *A. thaliana* (AP000423), *F. engleriana* (KX852398), *F. crenata* (MH171101), *F. sylvatica* (MK598696), and *F. japonica* var. *multinervis* (MN894556). Black circles represent GC content; green circles indicate G > C; purple circles indicate G < C.

The sequences from six cp genomes were compared using multigenome comparative analysis, employing the cp genome of *F. longipetiolata* as the reference genome (Figure 5). The cp genomes of these six plants (*A. thaliana*, *F. crenata*, *F. engleriana*, *F. japonica* var. *multinervis*, *F. longipetiolata*, and *F. sylvatica*) ranged in length from 154,478 bp to 158,462 bp, according to the findings. There was a high degree of similarity between *F. longipetiolata* and the other four *Fagus* cp genome sequences. They also had similarity with *A. thaliana* in IR regions (90~110 kbp and 130~155 kbp, Figure 5) but showed heterogeneity in other regions.

**Figure 6:**
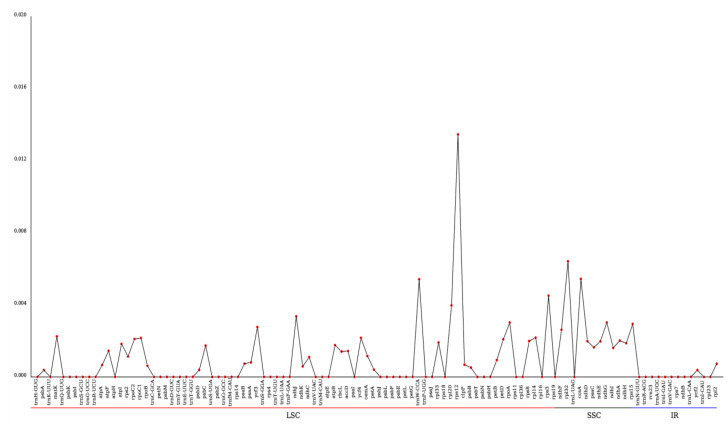
Line chart of gene Pi values calculated from 106 loci based on the five *Fagus* cp genomes. *x*-axis: gene name, *y*-axis: Pi value.

The nucleotide diversity (Pi) values of 106 loci in the chloroplast genome of *F. longipetiolata* glauca and four other *Fagus* plants (*F. engleriana*, *F. crenata*, *F. sylvatica,* and *F. japonica* var. *multinervis*) were calculated to determine the divergent hotspots (Figure 6). The minimum and maximum values of the entire genome sequence were between 0 and 0.01345, and the average value was 0.00099. The SSC area showed the maximum nucleotide diversity (average Pi = 0.00262), followed by the LSC region (average Pi = 0.00090), and the IR regions had the lowest Pi value (average Pi = 0.00009), indicating that the IR regions were substantially more conserved. In addition, five highly divergent regions were detected, including *rps12* (0.01345), *rpl32* (0.00641), *ccsA* (0.00543), *trnW-CCA* (0.00541), and *rps3* (0.00450). The LSC region contained three of these divergent regions (*rps12*, *trnW-CCA*, and *rps3*), and the SSC region contained two divergent regions (*rpl32* and *ccsA*).

### 3.5. IR Expansion and Contraction

**Figure 7:**
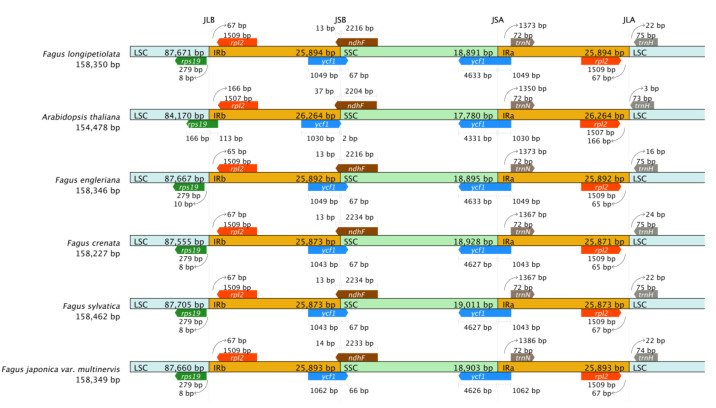
Comparisons of IR-SC boundary locations across the cp genomes of the five *Fagus* and *A. thaliana*.

We analyzed the binding regions of IR/LSC and IR/SSC of *F. longipetiolata* and five reference cp genomes (*F. sylvatica*, MK598696; *F. engleriana*, KX852398; *F. crenata*, MH171101; *F. japonica* var. *multinervis*, MN894556; *A. thaliana*, AP000423), as well as the length of genes located in the binding region. The genes located at the binding regions of the LSC/ IRB, IRB/SSC, SSC/IRA, and IRA/LSC regions were *rsp19*, *rpl2*, *ycf1*, *ndhF*, *ycf1*, *trnN*, *rpl2,* and *trnH*, respectively (Figure 7). The locations of the *rps19* genes of all *Fagus* plants were similar, occurring in the LSC region, 8–10 bp distant from the binding regions between the LSC and IRB, differing from the *rps19* gene location in *A. thaliana,* which spanned the LSC and IRB binding regions. This may be the reason that the *rpl2* gene of *A. thaliana* (166 bp) was farther from the LSC and IRB binding regions than that of *Fagus* (65–67 bp). The *trnH* gene was found to be located in the LSC region of *Fagus* species and 22 bp from the IRA/LSC boundary, except for F. engleriana (16 bp) and F. crenata (24 bp). The genes at IR-SSC junctions in all species were *ycf1* genes. The *ndhF* genes of all species were located mainly in the SSC region but also crossed the IRB/SSC boundary to some extent. The *ndhF* genes of *Fagus* extended into the IRB region by 13–14 bp, differing from that in *A. thaliana* by 37 bp. Based on the above results, the IRs and two SC regions of the five species of *Fagus*, in which the numbers and sequences of genes were conserved, showed slight differences at the borders.

### 3.6. Adaptive Evolution Analysis

**Figure 8:**
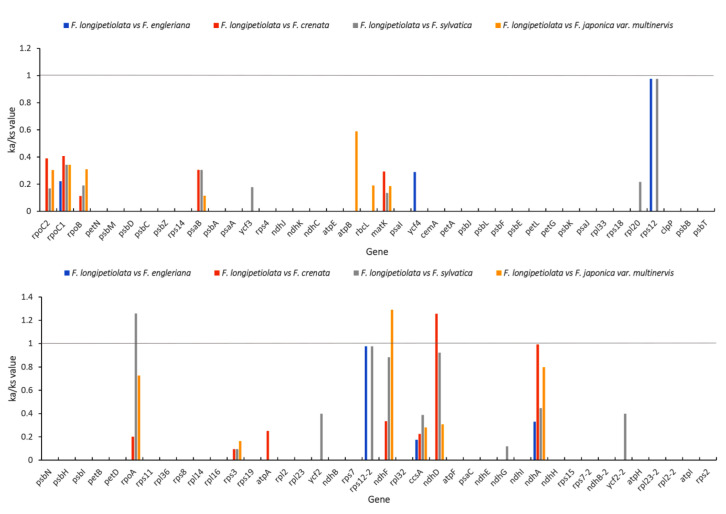
The 80 protein-coding genes of the *F. longipetiolata* cp genome and four *Fagus* species were used for ka/ka analysis.

Using *F. longipetiolata* as a reference, synonymous and nonsynonymous alterations in the five Fagus cp genomes were examined to uncover patterns of selection among protein-coding genes (Figure 8). In the five cp genomes, the Ka/Ks ratios of 80 protein-coding genes were determined by comparison. The ratio of Ka to Ks of most coding genes was less than one or could not be computed because one of the Ka or Ks values was zero, indicating that they were relatively conserved; in particular, the Ka/KS values of all the genes of *F. longipetiolata* and *F. engleriana* glauca were less than one. However, the Ka/Ks values of the *rpoA* gene between *F. longipetiolata* and *F. sylvatica*, the *ndhF* gene between *F. longipetiolata* and *F. japonica* var. *multinervis*, and the *ndhD* gene between *F. longipetiolata* and *F. crenata* were greater than one.

### 3.7. Phylogenetic Inference

**Figure 9:**
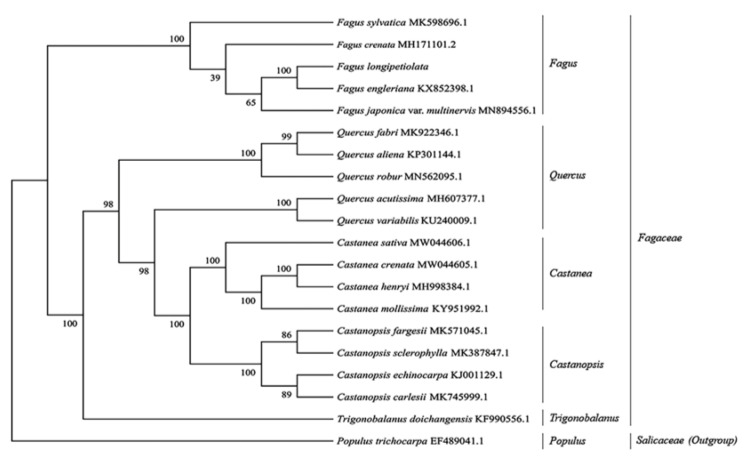
The phylogenetic tree was constructed by using the complete cp sequences of *F. longipetiolata* and 18 Fagaceae species. *P. trichocarpa* was used as the outgroup.

The cp genome is of great significance for system development research [36]. To determine the phylogenetic status of *F. longipetiolata* within Fagaceae, a phylogenetic tree was constructed using the ML method, using cp sequences of 18 Fagaceae species, with *Populus trichocarpa* as the outgroup (Figure 9). The analysis showed that the phylogenetic tree had a total of 18 nodes, 15 of which presented support rates ≥86%, and 10 presented support rates of 100%, which indicated that the reliability of the clustering results was high. In the phylogenetic tree, the 20 species of plants could be divided into two large groups and six small groups. *P. trichocarpa* of Salicaceae was located in one large group, and the 19 species of Fagaceae were in the other group. Moreover, the 19 Fagaceae species could be divided into *Fagus*, *Quercus*, *Castanea*, *Castanopsis,* and *Trigonobalanus*. Within the Fagaceae family, *Fagus* and *Trigonobalanus* were sister groups with high credibility. *F. longipetiolata* and *F. engleriana* were located on the same branch with 100% support, and this small branch belonged to the same branch as *F. japonica* var. *multinervis*. In addition, *F. sylvatica* was relatively distantly related to the other four *Fagus* species. This result shows that *F. longipetiolata* is highly homologous with *F. engleriana* but has a distant relationship with other plants in the genus.

## 4. Discussion

In general, the complete cp genome of *F. longipetiolata* showed great similarities to the other reported cp genomes of *Fagus* plants in terms of genome length, structure, and gene composition. No rearrangement phenomenon was observed, and a good collinearity relationship was found. Thus, the cp genome of *Fagus* is relatively well conserved [13,14]. Nevertheless, we observed minor differences in IR/SC border areas, which might be due to IR contraction and expansion. The contraction and expansion of the IR region is a common phenomenon in the process of evolution [37], and it is also the main reason for the differences in cp genome length [38]. However, the expansion and contraction of IR boundaries has not been shown to cause the transfer, gain or deletion of genes in the SC and IR regions of the cp genome, and consistent findings were obtained in the genomes of Fagaceae [13]. In angiosperms, the pseudogenes *ycf1* or *rps19* are produced by contraction and expansion of the IR region [39]. The *rps19* gene usually crosses the boundary between LSC/IR and SSC/IR [39,40]. In *Fagus*, the *rps19* coding gene was located in the LSC region, which is consistent with the results of other Fagaceae plants [41]. In this study, *ycf1* across the junction of IR/SSC, indicating that *ycf1* gene has no phylogenetic significance [42].

Nucleotide diversity (Pi) can indicate the magnitude of variation in various species’ nucleic acid sequences, and locations with higher variability can be used as molecular markers in population genetics [43,44]. In this study, the results of nucleotide diversity (Pi) assessment showed that the gene sequences of the LSC/SSC region were more variable than those in the IR region, which was consistent with the results found in other genera [13,41,45,46,47]. The same conclusion has been reached in the study of *Lagerstroemia* and *Adrinandra* plants [48,49]. Through cp genome sequence variation analysis, we discovered five hypervariable regions in the LSC (*rps12*, *trnW-CCA*, and *rps3*) and SSC regions (*rpl32* and *ccsA*). A previous study showed that point mutations in conserved regions of the *rps12* gene would affect the folding of 16S rRNA and the interaction with streptomycin in *Nicotiana plumbaginifolia* [50]. Moreover, the deletion/transfer of the *rpl32* gene in the plastid genome offers crucial phylogenetic data for the monophyletic evolution of the Thalictroideae subfamily [51]. It is also considered to be an ideal genetic marker for new *Glycine* varieties and *Diospyros* species [52,53]. The *ccsA* gene has been considered a locus to understand the evolution of the cp genome in *Litsea* [54], *Pterocarpus* [55], and *Prosopis* genera [56]. In this study, the Pi values of the five divergent regions were higher than 0.004, corresponding to highly variable regions. We suggest that the *rps12*, *rpl32*, *ccsA*, *trnW-CCA,* and *rps3* genes be used to study the molecular phylogeny of *Fagus*. They might be applicable for further analyses of phylogenetic relationships and population genetics and for species identification in *Fagus*.

The study of codon preference can not only aid in the interpretation of species evolution but can also be used to optimize the expression of foreign genes and to predict gene functions and gene expression levels [57]. In plant cp genomes, codons tend to end in an A or U base [58]. In this study, we found 33 high-frequency codons in *F. longipetiolata* dentata, 29 of which ended in A or U. This result may be caused by natural selection and mutation [59]. In addition, the amount of leucine was the highest and that of cysteine was the lowest among the amino acids. The same results were obtained in previous studies on the cp genomes of angiosperms [60].

In the evolution of species, as well as the inheritance and variation of genes within species, repeated sequences play a significant role [61,62]. In general, most of the repeated sequences in the genome are distributed in noncoding regions because of the process of species evolution. An organism retains the smallest amount of genetic information as much as possible to improve its genetic efficiency [63]. In this study, a total of 38 repeat sequences were discovered in the cp genome of *F. longipetiolata*, the majority of which were found in genes. It was indicated that the cp genome of *F. longipetiolata* retained abundant genetic information. SSRs of the plant plastid genome have been frequently employed in phylogenetic investigations on account of their unique maternal genetic advantages [64,65]. We found a total of 258 SSR sites in the cp genome of *F. longipetiolata*, among which single nucleotide repeats and trinucleotide repeats were the most common, with frequencies of 60.47 and 25.58%, respectively. These results were consistent with previous studies reported in the *F. crenata* and *F. engleriana* cp genomes [14]. It was suggested that the single nucleotide repetition of *Fagus* plants may play a more important role than other SSRs in genetic variation, which is similar to that of Lythraceae species [40]. In this study, the single nucleotides within the SSRs were almost A/T bases (95.51%), and AT/TA accounted for the majority of the two- to six-base repeats. *F. longipetiolata* had a relatively high A or T content and A/T polymerization at the SSR sites of cpDNA. These might be reasons for the high content of AT in the cp genome of *F. longipetiolata*, similar to the rich results of AT in other cp genomes [66,67]. Consistent with previous reports [68,69], the SSRs found in the cp genome of *F. longipetiolata* were mainly located in the LSC region and were enriched in the non-coding region. 

If a base mutation leads to an amino acid change, it is referred to as a nonsynonymous mutation; otherwise, it is a synonymous mutation, and nonsynonymous mutations are usually influenced by natural selection [70]. The selection effect of genes is usually expressed by the ratio of Ka to Ks. When Ka/Ks is greater than one, it indicates a positive selection effect, and when Ka/Ks is less than one, it indicates a purification selection effect [71]. In this study, the Ka/Ks of most genes (77 of 80) was less than one in the comparisons between *F. longipetiolata* and the other four *Fagus* species, showing that purifying selection plays an important role in the cp genes of the five species of *Fagus* species. However, in the three control groups, the Ka/Ks ratios of the *ndhD*, *rpoA,* and *ndhF* genes were greater than one, which showed that the three genes of *F. longipetiolata* were positively selected to adapt to the living environment. Positive selection of *ndhD* (*Pterocarpus* and Leguminosae) [50,72], *rpoA* (*Trifolium alexandrinum* and *Trifolium resupinatum*), and *ndhF* (*T. alexandrinum*, *T. resupinatum* and *Cardamine* genus) [73,74] genes has also been reported in previous studies. It is also reported that NADPH dehydrogenase genes (*ndhD* and *ndhA*) tend to evolve at a higher rate than other genes [75]. The low Ka/Ks ratio (ka/ks = 0) of the ClpP gene in this study may be due to the fact that the *ClpP* gene contains two introns, which is similar to that of Zingibereae [42]. The evolution rate of the *ClpP* gene is species-specific, which would lead to the loss of introns in the process of rapid evolution [76]. 

Here, phylogenetic analysis relying on the cp genome revealed that *Fagus* is a sister genus of all other Fagaceae plants and forms a monophyletic branch, which was similar to the results of previous studies [15,16,77]. Research using fossil records also suggests that the *Fagus* is closer to the ancestral group of Fagaceae than *Quercus* [78]. We concluded that *Fagus* and *Trigonobalanus* were located at the base of the phylogenetic tree (Figure 9), which was consistent with the fossil records [78]. An earlier study on the cp genome of *Quercus* also obtained similar results, although only one of the cp genomes (*F. engleriana*) was used in *Fagus* [41]. In this study, we showed that *F. longipetiolata* was closely related to *F. engleriana*. Moreover, they are sympatric, with distributions partially overlapping in geographical areas [17]. It indicates potential introgressive effects in these two species. However, the cp genomes of a number of species within *Fagus* have not yet been published. Further research of the *Fagus* cp genomes may provide more evidence to clarify the relationship between chloroplast phylogeny and geographic distribution.

## 5. Conclusions

In this study, we published the complete cp genome sequence of *F. longipetiolata* for the first time and compared it with those of other *Fagus* species, providing a useful reference for the phylogeny of *Fagus*. Although the cp genomes of *F. longipetiolata* and other *Fagus* were substantially similar in terms of genome structure, gene content, and gene sequences, some hot spots could be found in LSC and SSC regions, which would provide informative markers for the phylogenetic analysis of *Fagus*. The analysis of the selection pressure on the *Fagus* cp genome showed that the *ndhD*, *rpoA*, and *ndhF* genes of *F. longipetiolata* were affected by positive choices. Phylogenetic research showed a tight connection between *F. longipetiolata* and *F. engleriana*, which partially overlapped in their distribution in China. The phylogenetic relationship of *Fagus* with Fagaceae has been well resolved and strongly supports that *Fagus* is a monophyletic group. The new genome information obtained in this study can only contribute to the better development and utilization of *F. longipetiolata* but also provide reference data for population genome research, phylogenetic analysis and genetic engineering research.

## Figures and Tables

**Table 1:** Characteristics of *F. longipetiolata* cp genome.

Category	Item	Describe
Chloroplast genome structure	Cp gene/bp	158,350
	LSC/bp	87,671
	SSC/bp	18,891
	IRA/IRB/bp	25,894
Gene composition	Cp gene	131
	CDS	81
	tRNA	37
	rRNA	8
	pseudo	5
GC Content (%)	Cp gene	37.09
	LSC	35.05
	SSC	31.19
	IRA/IRB	42.70

**Table 2:** Genes in cp genome of *F. longipetiolata*.

Category	Gene Group	Gene Name
Photosynthesis	Subunits of photosystem I	psaA, psaB, psaC, psaI, psaJ
	Subunits of photosystem II	psbA, psbB, psbC, psbD, psbE, psbF, psbH, psbI, psbJ, psbK, psbL, psbM, psbN, psbT, psbZ
	Subunits of NADH dehydrogenase	ndhA *, ndhB * (2), ndhC, ndhD, ndhE, ndhF, ndhG, ndhH, ndhI, ndhJ, ndhK
	Subunits of cytochrome b/f complex	petA, petB *, petD *, petG, petL, petN
	Subunits of ATP synthase	atpA, atpB, atpE, atpF *, atpH, atpI
	Large subunit of rubisco	rbcL
	Subunits photochlorophyllide reductase	-
Self-replication	Proteins of large ribosomal subunit	# rpl22, rpl14, rpl16 *, rpl2 * (2), rpl20, rpl23 (2), rpl32, rpl33, rpl36
	Proteins of small ribosomal subunit	# rps16, rps11, rps12 * (2), rps14, rps15, rps18, rps19, rps2, rps3, rps4, rps7 (2), rps8
	Subunits of RNA polymerase	rpoA, rpoB, rpoC1 *, rpoC2
	Ribosomal RNAs	rrn16 (2), rrn23 (2), rrn4.5 (2), rrn5 (2)
	Transfer RNAs	trnA-UGC * (2), trnC-GCA, trnD-GUC, trnE-UUC, trnF-GAA, trnG-GCC, trnG-UCC *, trnH-GUG, trnI-CAU (2), trnI-GAU * (2), trnK-UUU *, trnL-CAA (2), trnL-UAA *, trnL-UAG, trnM-CAU, trnN-GUU (2), trnP-UGG, trnQ-UUG, trnR-ACG (2), trnR-UCU, trnS-GCU, trnS-GGA, trnS-UGA, trnT-GGU, trnT-UGU, trnV-GAC (2), trnV-UAC *, trnW-CCA, trnY-GUA, trnfM-CAU
Other genes	Maturase	matK
	Protease	clpP **
	Envelope membrane protein	cemA
	Acetyl-CoA carboxylase	accD
	c-type cytochrome synthesis gene	ccsA
	Translation initiation factor	# infA
	other	-
Genes of unknown function	Conserved hypothetical chloroplast ORF	# ycf1 (2), ycf2 (2), ycf3 **, ycf4

Note: # Gene, Pseudo gene; Gene (2), Multiple copy gene, the number of copies in parenthesis; Gene *, Gene with one intron; Gene **, Genes containing two introns.

## Data Availability

The data presented in this study are available in the article and Supplementary Materials. The whole chloroplast genomes data of this study are openly available in GenBank of NCBI at https://www.ncbi.nlm.nih.gov (accessed on 13 June 2021) under accession number: MZ562567. The associated BioProject, BioSample and SRA numbers are PRJNA792394, SAMN24423804 and SRX13508090, respectively.

## Supplementary Materials

The following supporting information can be downloaded at: www.mdpi.com/article/10.3390/life12010092/s1, Table S1: The lengths of the introns and exons of the genes of the *F. longipetiolata* cp genome, Table S2: Codon usage and amino acid type statistics of the cp genome of *F. longipetiolata*, Table S3: Repeated sequences in the *F. longipetiolata* cp genome, Table S4: Simple sequence repeats (SSRs) in the *F. longipetiolata* cp genome.
