# Supplementary material for: Complete Chloroplast Genome Sequence of Fagus longipetiolata Seemen (Fagaceae): Genome Structure, Adaptive Evolution, and Phylogenetic Relationships
Source: Life (Basel). 2022 Jan 9;12(1):92. doi: 10.3390/life12010092 (PMC8778281; doi:10.3390/life12010092)
Supplement: Supplementary file 1 [file life-12-00092-s001.zip › life-1502840-supplementary/Supplementary Materials/Table S1.pdf]

Table S1. The lengths of introns and exons in genes in the *F. longipetiolata* chloroplast genome.

Gene	Location	Exon I (bp)	Intron I (bp)	Exon II (bp)	Intron II bp)	Exon III (bp)
trnK-UUU	LSC	37	2524	35		
trnG-UCC	LSC	23	724	48		
atpF	LSC	145	762	410		
rpoC1	LSC	453	795	1617		
ycf3	LSC	124	773	230	718	153
trnL-UAA	LSC	35	535	50		
trnV-UAC	LSC	38	622	35		
rps12	IRa	114	-	232	538	26
clpP	LSC	71	639	292	879	228
petB	LSC	6	800	642		
petD	LSC	8	620	475		
rpl16	LSC	9	1089	399		
rpl2	IRb	391	684	434		
ndhB	IRb	777	681	756		
rps12	IRb	232	-	26	538	114
trnI-GAU	IRb	37	955	35		
trnA-UGC	IRb	38	804	35		
ndhA	SSC	553	1068	545		
trnA-UGC	IRa	38	804	35		
trnI-GAU	IRa	37	955	35		
ndhB	IRa	777	681	756		
rpl2	IRa	391	684	434		
