# Supplementary material for: Complete Chloroplast Genome Sequence of Fagus longipetiolata Seemen (Fagaceae): Genome Structure, Adaptive Evolution, and Phylogenetic Relationships
Source: Life (Basel). 2022 Jan 9;12(1):92. doi: 10.3390/life12010092 (PMC8778281; doi:10.3390/life12010092)
Supplement: Supplementary file 1 [file life-12-00092-s001.zip › life-1502840-supplementary/Supplementary Materials/Table S2.pdf]

Table S2. Codon usage and amino acid type statistics of chloroplast genome of *F. longipetiolata*

AminoAcid	Symbol	Codon	No	Password sub proportion /%	Number of amino acids	Amino acid ratio /%	RSCU
*	Ter	UAA	45	0.19%	81	0.34%	1.6668
*	Ter	UAG	20	0.08%			0.7407
*	Ter	UGA	16	0.07%			0.5925
A	Ala	GCA	366	1.51%	1340	5.54%	1.0924
A	Ala	GCC	217	0.90%			0.6476
A	Ala	GCG	157	0.65%			0.4688
A	Ala	GCU	600	2.48%			1.7912
C	Cys	UGC	78	0.32%	276	1.14%	0.5652
C	Cys	UGU	198	0.82%			1.4348
D	Asp	GAC	199	0.82%	967	4.00%	0.4116
D	Asp	GAU	768	3.18%			1.5884
E	Glu	GAA	906	3.75%	1233	5.10%	1.4696
E	Glu	GAG	327	1.35%			0.5304
F	Phe	UUC	503	2.08%	1378	5.70%	0.73
F	Phe	UUU	875	3.62%			1.27
G	Gly	GGA	663	2.74%	1709	7.07%	1.5516
G	Gly	GGC	189	0.78%			0.4424
G	Gly	GGG	296	1.22%			0.6928
G	Gly	GGU	561	2.32%			1.3132
H	His	CAC	142	0.59%	590	2.44%	0.4814
H	His	CAU	448	1.85%			1.5186
I	Ile	AUA	683	2.83%	2108	8.72%	0.972
I	Ile	AUC	405	1.68%			0.5763
I	Ile	AUU	1020	4.22%			1.4517
K	Lys	AAA	847	3.50%	1165	4.82%	1.454
K	Lys	AAG	318	1.32%			0.546
L	Leu	CUA	345	1.43%	2553	10.56%	0.8106
L	Leu	CUC	180	0.74%			0.423
L	Leu	CUG	174	0.72%			0.4092
L	Leu	CUU	546	2.26%			1.2834
L	Leu	UUA	779	3.22%			1.8306
L	Leu	UUG	529	2.19%			1.2432
M	Met	AUA	1	0.00%	559	2.31%	0.0054
M	Met	AUG	557	2.30%			2.9892
M	Met	GUG	1	0.00%			0.0054
N	Asn	AAC	275	1.14%	1146	4.74%	0.48
N	Asn	AAU	871	3.60%			1.52
P	Pro	CCA	285	1.18%	1028	4.25%	1.1088
P	Pro	CCC	194	0.80%			0.7548
P	Pro	CCG	158	0.65%			0.6148
P	Pro	CCU	391	1.62%			1.5216
Q	Gln	CAA	660	2.73%	862	3.57%	1.5314
Q	Gln	CAG	202	0.84%			0.4686
R	Arg	AGA	419	1.73%	1446	5.98%	1.7388
R	Arg	AGG	160	0.66%			0.6642
R	Arg	CGA	340	1.41%			1.4106
R	Arg	CGC	96	0.40%			0.3984
R	Arg	CGG	106	0.44%			0.4398
R	Arg	CGU	325	1.34%			1.3488
S	Ser	AGC	115	0.48%	1863	7.71%	0.3702
S	Ser	AGU	358	1.48%			1.1532
S	Ser	UCA	355	1.47%			1.1436
S	Ser	UCC	317	1.31%			1.0212
S	Ser	UCG	180	0.74%			0.5796
S	Ser	UCU	538	2.23%			1.7328
T	Thr	ACA	358	1.48%	1216	5.03%	1.1776
T	Thr	ACC	229	0.95%			0.7532
T	Thr	ACG	140	0.58%			0.4604
T	Thr	ACU	489	2.02%			1.6084
V	Val	GUA	519	2.15%	1332	5.51%	1.5584
V	Val	GUC	162	0.67%			0.4864
V	Val	GUG	183	0.76%			0.5496
V	Val	GUU	468	1.94%			1.4056
W	Trp	UGG	428	1.77%	428	1.77%	1
Y	Tyr	UAC	184	0.76%	889	3.68%	0.414

Y	Tyr	UAU	705	2.92%	1.586
