# Supplementary material for: Complete Chloroplast Genome Sequence of Fagus longipetiolata Seemen (Fagaceae): Genome Structure, Adaptive Evolution, and Phylogenetic Relationships
Source: Life (Basel). 2022 Jan 9;12(1):92. doi: 10.3390/life12010092 (PMC8778281; doi:10.3390/life12010092)
Supplement: Supplementary file 1 [file life-12-00092-s001.zip › life-1502840-supplementary/Supplementary Materials/Table S3.pdf]

Table S3. Repeated sequences of the *F. longipetiolata* chloroplast genome.

ID	Length I	Repeat I Start	Type	Length II	Repeat II Start	Hamming distance	E-value	Gene	Region
1	46	33060	P	46	33060	0	1.42E-18	IGS	LSC;LSC
2	44	77633	P	44	77633	-2	1.94E-13	IGS	LSC;LSC
3	43	49726	F	43	49769	0	9.11E-17	IGS	LSC;LSC
4	42	46568	F	42	101950	-3	1.13E-10	ycf3;IGS	LSC;IRb
5	42	46568	P	42	144031	-3	1.13E-10	ycf3;IGS	LSC;IRa
6	40	101948	F	40	124264	-1	7.00E-13	IGS;ndhA	IRb;SSC
7	40	124264	P	40	144035	-1	7.00E-13	ndhA;IGS	SSC;IRa
8	38	13406	P	38	13406	0	9.33E-14	IGS	LSC;LSC
9	38	46568	F	38	124266	-1	1.06E-11	ycf3;ndhA	LSC;SSC
10	36	7917	P	36	48217	-3	2.88E-07	trnS-GCU;trnS-GGA	LSC;LSC
11	35	78991	P	35	124267	-3	1.06E-06	petB;ndhA	LSC;SSC
12	34	13377	P	34	13377	-2	1.21E-07	IGS	LSC;LSC
13	34	131681	P	34	131681	-2	1.21E-07	ycf1;ycf1	SSC;SSC
14	33	41548	F	33	43772	-3	1.41E-05	psaB;psaA	LSC;LSC
15	33	84962	F	33	124255	-3	1.41E-05	rpl16;ndhA	LSC;SSC
16	33	73951	R	33	73955	-3	1.41E-05	clpP;clpP	LSC;LSC
17	32	92485	F	32	92506	-3	5.12E-05	ycf2;ycf2	IRb;IRb
18	32	92485	P	32	153485	-3	5.12E-05	ycf2;ycf2	IRb;IRa
19	32	92506	P	32	153506	-3	5.12E-05	ycf2;ycf2	IRb;IRa
20	32	153485	F	32	153506	-3	5.12E-05	ycf2;ycf2	IRa;IRa
21	32	51860	C	32	68575	-3	5.12E-05	IGS	LSC;LSC
22	31	7912	F	31	38127	-3	1.86E-04	trnS-GCU;trnS-UGA	LSC;LSC
23	31	68387	P	31	68387	-3	1.86E-04	IGS	LSC;LSC
24	31	84971	F	31	124264	-3	1.86E-04	rpl16;ndhA	LSC;SSC
25	30	110882	F	30	110913	-1	5.51E-07	IGS	IRb;IRb
26	30	110882	P	30	135080	-1	5.51E-07	IGS	IRb;IRa
27	30	110913	P	30	135111	-1	5.51E-07	IGS	IRb;IRa
28	30	135080	F	30	135111	-1	5.51E-07	IGS	IRa;IRa
29	30	46580	F	30	101962	-2	2.39E-05	ycf3;IGS	LSC;IRb
30	30	46580	P	30	144031	-2	2.39E-05	ycf3;IGS	LSC;IRa
31	30	38131	P	30	48224	-3	6.71E-04	trnS-UGA;trnS-GGA	LSC;LSC
32	30	73955	P	30	102162	-3	6.71E-04	clpP;IGS	LSC;IRb
33	30	73955	F	30	143831	-3	6.71E-04	clpP;IGS	LSC;IRa
34	30	73953	R	30	73958	-2	2.39E-05	clpP;clpP	LSC;LSC
35	30	49911	R	30	49917	-3	6.71E-04	IGS	LSC;LSC
36	30	49911	R	30	49913	-3	6.71E-04	IGS	LSC;LSC
37	30	74592	R	30	74595	-3	6.71E-04	clpP;clpP	LSC;LSC

Note: Type represents the type of repeat sequence, F stands for forward repetition, P for palindromic repetition, R for reverse repetition, and C for complement repe-tition; IGS represents the intergenic space.
