# Supplementary material for: Complete Chloroplast Genome Sequence of Fagus longipetiolata Seemen (Fagaceae): Genome Structure, Adaptive Evolution, and Phylogenetic Relationships
Source: Life (Basel). 2022 Jan 9;12(1):92. doi: 10.3390/life12010092 (PMC8778281; doi:10.3390/life12010092)
Supplement: Supplementary file 1 [file life-12-00092-s001.zip › life-1502840-supplementary/Supplementary Materials/Table S4.pdf]

Table S4. Simple sequence repeats (SSRs) in the *F. longipetiolata* chloroplast genome.

ID	Repeat Motif	Length(bp)	Start	End	Region	Gene	ID	Repeat Motif	Length(bp)	Start	End	Region	Gene
1	(A)10	10	172	181	LSC		130	(T)8	8	63639	63646	LSC	
2	(T)10	10	217	226	LSC		131	(T)11	11	63715	63725	LSC	
3	(A)8	8	255	262	LSC		132	(TC)5	10	64166	64175	LSC	cemA
4	(A)8	8	278	285	LSC		133	(C)8	8	65374	65381	LSC	petA
5	(AT)6	12	1706	1717	LSC		134	(A)8	8	65448	65455	LSC	petA
6	(T)10	10	1744	1753	LSC		135	(T)8	8	66094	66101	LSC	
7	(A)12	12	1772	1783	LSC		136	(T)15	15	66186	66200	LSC	
8	(T)9	9	2393	2401	LSC	matK	137	(AAT)3	9	66351	66359	LSC	
9	(TAA)3	9	3084	3092	LSC	matK	138	(AT)5	10	66548	66557	LSC	
10	(A)10	10	3914	3923	LSC	trnK-UUU	139	(A)9	9	66584	66592	LSC	
11	(A)9	9	4005	4013	LSC	trnK-UUU	140	(GTA)3	9	67964	67972	LSC	
12	(T)8	8	4197	4204	LSC	trnK-UUU	141	(T)10	10	68167	68176	LSC	
13	(A)10	10	4433	4442	LSC		142	(A)11gatc(A)9	24	68575	68598	LSC	
14	(A)8	8	4538	4545	LSC		143	(A)8	8	69113	69120	LSC	
15	(A)10	10	4687	4696	LSC		144	(A)8	8	70031	70038	LSC	
16	(A)17	17	4750	4766	LSC		145	(T)10	10	70078	70087	LSC	
17	(TA)5	10	4985	4994	LSC		146	(TTA)3	9	70696	70704	LSC	
18	(A)8	8	5086	5093	LSC		147	(A)9	9	71140	71148	LSC	
19	(T)9	9	5112	5120	LSC		148	(AAC)3	9	71366	71374	LSC	rps18
20	(C)13(A)9	22	5500	5521	LSC		149	(A)9	9	71617	71625	LSC	
21	(T)9	9	5876	5884	LSC		150	(T)10	10	72316	72325	LSC	
22	(T)9	9	5955	5963	LSC		151	(AAT)3	9	73530	73538	LSC	clpP
23	(ATA)3	9	6127	6135	LSC		152	(ATTTT)3	15	73701	73715	LSC	clpP
24	(T)8	8	6211	6218	LSC		153	(A)14t(A)12	27	73956	73982	LSC	clpP
25	(A)9	9	6423	6431	LSC		154	(A)9	9	74460	74468	LSC	clpP
26	(A)9	9	7289	7297	LSC		155	(AT)8	16	74600	74615	LSC	clpP
27	(A)8	8	7852	7859	LSC		156	(T)11	11	74827	74837	LSC	clpP
28	(AT)5	10	8240	8249	LSC		157	(T)9	9	75750	75758	LSC	
29	(TAA)3	9	8337	8345	LSC		158	(T)8gccgctt(TTG)3	24	76550	76573	LSC	psbB
30	(TAA)3	9	8379	8387	LSC		159	(TCT)3	9	77198	77206	LSC	psbB
31	(TTG)3	9	8553	8561	LSC		160	(T)8	8	78203	78210	LSC	
32	(A)9	9	8669	8677	LSC		161	(A)8	8	78445	78452	LSC	petB
33	(A)9	9	9142	9150	LSC	trnG-UCC	162	(A)8	8	81195	81202	LSC	
34	(A)11	11	11898	11908	LSC		163	(TTC)3	9	81518	81526	LSC	rpoA
35	(GTT)3	9	12081	12089	LSC	atpF	164	(ATA)3	9	82221	82229	LSC	rpoA
36	(T)9	9	12464	12472	LSC	atpF	165	(T)8	8	82785	82792	LSC	
37	(T)12	12	12598	12609	LSC	atpF	166	(A)10	10	83060	83069	LSC	
38	(CAAT)3	12	12699	12710	LSC	atpF	167	(T)8	8	83883	83890	LSC	
39	(ATT)4	12	12902	12913	LSC	atpF	168	(CAA)3	9	84271	84279	LSC	rpl14
40	(A)10	10	13044	13053	LSC	atpF	169	(A)8	8	84433	84440	LSC	
41	(A)8	8	13114	13121	LSC	atpF	170	(A)8	8	84469	84476	LSC	
42	(A)8	8	13670	13677	LSC		171	(T)9	9	84491	84499	LSC	
43	(TAA)3	9	14104	14112	LSC		172	(T)17	17	85246	85262	LSC	rpl16
44	(T)8	8	14247	14254	LSC		173	(A)8	8	86049	86056	LSC	
45	(CCTCC)4	20	14480	14499	LSC		174	(TTG)3	9	87154	87162	LSC	
46	(T)9	9	14513	14521	LSC		175	(TTA)3	9	87567	87575	LSC	rps19
47	(T)13	13	14548	14560	LSC		176	(T)9	9	87641	87649	LSC	rps19
48	(T)8	8	14756	14763	LSC		177	(T)8	8	87693	87700	IR	
49	(AAC)3	9	15247	15255	LSC	atpI	178	(CTT)3	9	87794	87802	IR	rpl2
50	(A)8	8	15890	15897	LSC		179	(GAT)3	9	88259	88267	IR	rpl2
51	(ATTT)3(TTA)3	21	16065	16085	LSC		180	(A)9	9	93109	93117	IR	ycf2
52	(A)8	8	16830	16837	LSC		181	(TCT)3	9	93813	93821	IR	ycf2
53	(A)11	11	16982	16992	LSC		182	(AAT)4	12	93844	93855	IR	ycf2
54	(T)9	9	18948	18956	LSC	rpoC2	183	(CTT)3	9	93925	93933	IR	ycf2
55	(T)10	10	19055	19064	LSC	rpoC2	184	(GAA)3	9	95637	95645	IR	ycf2
56	(A)8	8	19197	19204	LSC	rpoC2	185	(AGA)3	9	98694	98702	IR	ndhB
57	(AGC)3	9	19419	19427	LSC	rpoC2	186	(T)8	8	98927	98934	IR	ndhB
58	(T)12	12	19583	19594	LSC	rpoC2	187	(AGA)3	9	100134	100142	IR	ndhB
59	(AT)5	10	20424	20433	LSC	rpoC2	188	(A)9	9	100296	100304	IR	
60	(TTC)3	9	22693	22701	LSC	rpoC1	189	(T)8	8	102139	102146	IR	
61	(A)9	9	23514	23522	LSC	rpoC1	190	(GCAAT)3	15	102882	102896	IR	
62	(T)10	10	23551	23560	LSC	rpoC1	191	(AAG)3	9	103229	103237	IR	
63	(T)8	8	26813	26820	LSC	rpoB	192	(T)8	8	106173	106180	IR	trnI-GAU
64	(C)8	8	26989	26996	LSC	rpoB	193	(G)9	9	107396	107404	IR	trnA-UGC
65	(T)8	8	27883	27890	LSC		194	(CTG)3	9	107954	107962	IR	rrn23

66	(T)8	8	27962	27969	LSC		195	(A)8	8	111256	111263	IR	
67	(C)10	10	28657	28666	LSC		196	(AG)5	10	111344	111353	IR	
68	(A)8	8	28793	28800	LSC		197	(T)10	10	111403	111412	IR	
69	(G)8	8	28865	28872	LSC		198	(CAA)3	9	112996	113004	IR	
70	(CTA)3	9	29842	29850	LSC		199	(TCCTTC)3	18	113356	113373	IR	
71	(T)8	8	29957	29964	LSC		200	(A)10	10	113816	113825	SSC	ndhF
72	(T)10	10	30802	30811	LSC		201	(A)8	8	114254	114261	SSC	ndhF
73	(T)8	8	31540	31547	LSC		202	(T)9	9	114311	114319	SSC	ndhF
74	(AAAT)3	12	31972	31983	LSC		203	(TAA)3	9	115209	115217	SSC	ndhF
75	(A)11	11	32381	32391	LSC		204	(T)8	8	115842	115849	SSC	
76	(A)10	10	33304	33313	LSC		205	(A)9gtgaaa(AT)5	25	115961	115985	SSC	
77	(AAT)3	9	34003	34011	LSC		206	(T)8	8	116254	116261	SSC	
78	(TAT)3	9	34089	34097	LSC		207	(A)8	8	116549	116556	SSC	
79	(TTA)3	9	34161	34169	LSC		208	(T)12	12	117652	117663	SSC	
80	(T)12	12	34386	34397	LSC		209	(ATT)3	9	117924	117932	SSC	
81	(TTA)3	9	34548	34556	LSC		210	(A)8	8	118513	118520	SSC	ccsA
82	(A)9	9	34790	34798	LSC		211	(T)8	8	120106	120113	SSC	ndhD
83	(A)9	9	34854	34862	LSC		212	(T)9	9	121083	121091	SSC	ndhD
84	(A)9	9	35257	35265	LSC		213	(A)8	8	121120	121127	SSC	ndhD
85	(GGA)3	9	37039	37047	LSC	psbC	214	(T)10	10	122776	122785	SSC	
86	(TTGG)3	12	37941	37952	LSC		215	(AAAG)3	12	123002	123013	SSC	
87	(T)8	8	38017	38024	LSC		216	(TTC)3	9	124062	124070	SSC	ndhA
88	(TA)5	10	38350	38359	LSC		217	(A)10gatcta(T)8	24	124571	124594	SSC	ndhA
89	(A)10	10	38393	38402	LSC		218	(A)9(G)8	17	124651	124667	SSC	ndhA
90	(TTA)4	12	39037	39048	LSC		219	(TTC)3	9	125036	125044	SSC	ndhA
91	(A)8	8	39186	39193	LSC		220	(AT)6	12	125114	125125	SSC	ndhA
92	(A)8	8	39518	39525	LSC		221	(AGC)3	9	125349	125357	SSC	ndhA
93	(TCT)3	9	40024	40032	LSC	rps14	222	(T)9	9	127423	127431	SSC	
94	(ATG)3	9	41560	41568	LSC	psaB	223	(TAA)3	9	127465	127473	SSC	
95	(TTG)3	9	43165	43173	LSC	psaA	224	(AAT)3	9	127565	127573	SSC	
96	(GCA)3	9	43458	43466	LSC	psaA	225	(A)8	8	127643	127650	SSC	
97	(T)8	8	45671	45678	LSC	ycf3	226	(A)8	8	127809	127816	SSC	
98	(T)14	14	47164	47177	LSC	ycf3	227	(A)8	8	128856	128863	SSC	
99	(A)8tcatt(A)9	22	47462	47483	LSC		228	(T)10	10	129001	129010	SSC	
100	(A)9	9	47979	47987	LSC		229	(T)8	8	130004	130011	SSC	
101	(A)8	8	49315	49322	LSC		230	(T)9	9	130316	130324	SSC	
102	(A)18	18	49920	49937	LSC		231	(T)8	8	130493	130500	SSC	
103	(A)10	10	50216	50225	LSC		232	(T)9aattttaa(T)11	28	130600	130627	SSC	
104	(A)10	10	50553	50562	LSC	trnL-UAA	233	(T)9	9	131199	131207	SSC	
105	(TTTA)3	12	50736	50747	LSC	trnL-UAA	234	(A)8	8	131258	131265	SSC	
106	(TAAAA)3	15	51423	51437	LSC		235	(A)9	9	131971	131979	SSC	
107	(T)13	13	51858	51870	LSC		236	(GAGAAG)3	18	132647	132664	IR	
108	(A)10	10	52688	52697	LSC		237	(TTG)3	9	133018	133026	IR	
109	(AAC)3	9	53258	53266	LSC	ndhK	238	(A)10	10	134610	134619	IR	
110	(T)9a(CTTT)3	22	53410	53431	LSC		239	(CT)5	10	134669	134678	IR	
111	(AT)6	12	54004	54015	LSC		240	(T)8	8	134759	134766	IR	
112	(T)9	9	54080	54088	LSC		241	(CAG)3	9	138060	138068	IR	rrn23
113	(TTA)3	9	54164	54172	LSC		242	(C)9	9	138618	138626	IR	trnA-UGC
114	(T)9	9	54645	54653	LSC	trnV-UAC	243	(A)8	8	139842	139849	IR	trnI-GAU
115	(T)9attgaaa(T)10	26	55294	55319	LSC		244	(CTT)3	9	142785	142793	IR	
116	(T)10	10	57233	57242	LSC	atpB	245	(CATTG)3	15	143125	143139	IR	
117	(T)9	9	57693	57701	LSC		246	(A)8	8	143876	143883	IR	
118	(A)8	8	57826	57833	LSC		247	(T)9	9	145718	145726	IR	
119	(TTG)3	9	58031	58039	LSC		248	(TTC)3	9	145879	145887	IR	ndhB
120	(GCT)3	9	59442	59450	LSC	rbcL	249	(A)8	8	147088	147095	IR	ndhB
121	(T)8	8	59928	59935	LSC		250	(TCT)3	9	147320	147328	IR	ndhB
122	(T)8	8	59952	59959	LSC		251	(TTC)3	9	150377	150385	IR	ycf2
123	(G)8	8	60138	60145	LSC	accD	252	(AAG)3	9	152089	152097	IR	ycf2
124	(T)8	8	61629	61636	LSC		253	(ATT)4	12	152167	152178	IR	ycf2
125	(T)8	8	61754	61761	LSC		254	(AGA)3	9	152201	152209	IR	ycf2
126	(A)11	11	61805	61815	LSC		255	(T)9	9	152905	152913	IR	ycf2
127	(T)8	8	62487	62494	LSC		256	(ATC)3	9	157755	157763	IR	rpl2
128	(T)8	8	63052	63059	LSC	ycf4	257	(GAA)3	9	158219	158227	IR	rpl2
129	(T)9	9	63527	63535	LSC		258	(A)8	8	158322	158329	IR	
